# Investigation of Long-Term CD4+ T Cell Receptor Repertoire Changes Following SARS-CoV-2 Infection in Patients with Different Severities of Disease

**DOI:** 10.3390/diagnostics14202330

**Published:** 2024-10-19

**Authors:** Emma L. Callery, Camilo L. M. Morais, Jemma V. Taylor, Kirsty Challen, Anthony W. Rowbottom

**Affiliations:** 1 Department of Immunology, Lancashire Teaching Hospitals NHS Foundation, Preston PR2 9HT, UK; jemma.taylor@lthtr.nhs.uk; 2 Institute of Chemistry, Federal University of Rio Grande do Norte, Natal 59072-970, Brazil; camilomorais1@gmail.com; 3 Department of Emergency Medicine, Lancashire Teaching Hospitals NHS Foundation, Preston PR2 9HT, UK; kirsty.challen@lthtr.nhs.uk; 4 School of Medicine, University of Central Lancashire, Preston PR1 2HE, UK

**Keywords:** COVID-19, severity model, machine learning, T cell receptor, TCR repertoire, immune response, flow cytometry, SARS-CoV-2

## Abstract

Background: The difference in the immune response to severe acute respiratory syndrome coro-navirus 2 (SARS-CoV-2) in patients with mild versus severe disease remains poorly understood. Recent scientific advances have recognised the vital role of both B cells and T cells; however, many questions remain unanswered, particularly for T cell responses. T cells are essential for helping the generation of SARS-CoV-2 antibody responses but have also been recognised in their own right as a major factor influencing COVID-19 disease outcomes. The examination of T cell receptor (TCR) family differences over a 12-month period in patients with varying COVID-19 disease severity is crucial for understanding T cell responses to SARS-CoV-2. Methods: We applied a machine learning approach to analyse TCR vb family responses in COVID-19 patients (*n* = 151) across multiple timepoints and disease severities alongside SARS-CoV-2 infection-naïve (healthy control) individ-uals (*n* = 62). Results: Blood samples from hospital in-patients with moderate, severe, or critical disease could be classified with an accuracy of 94%. Furthermore, we identified significant variances in TCR vb family specificities between disease and control subgroups. Conclusions: Our findings suggest advantageous and disadvantageous TCR repertoire patterns in relation to disease severity. Following validation in larger cohorts, our methodology may be useful in detecting protective immunity and the assessment of long-term outcomes, particularly as we begin to unravel the immunological mechanisms leading to post-COVID complications.

## 1. Introduction

The COVID-19 pandemic led to approximately 7.01 million deaths worldwide as of 9th April 2024. Disease severity varied significantly among individuals, from asymptomatic or very mild symptoms to lethal disease. Whilst many different demographic factors, such as gender, ethnicity, age, and comorbidities, have been shown to affect disease outcome, they are unable to completely account for differences in mortality rates around the world [1,2,3]. The adaptive immune response is essential for viral clearance in COVID-19 disease, with several recent studies highlighting the significant contribution of the T cell receptor (TCR) repertoire on the course of SARS-CoV-2 infection [4].

In response to the pandemic, an unprecedented international research effort in essentially all aspects of SARS-CoV-2 biology was initiated, with high-efficacy vaccines and antivirals produced in record time. Recent scientific advances have begun to shed light on the long-term effects of SARS-CoV-2 infection, including prolonged dysregulation of the immune system. Recently, a definition for long COVID was developed by the National Academy for Science Engineering and Medicine (NASEM): ‘Long COVID (LC) is an infection-associated chronic condition (IACC) that occurs after SARS-CoV-2 infection and is present for at least 3 months as a continuous, relapsing and remitting, or progressive disease state that affects one or more organ systems’ [5]. The aim of the consensus report is to overcome challenges faced by policymakers, researchers, public health professionals, clinicians, and support services, and to improve patient management.

Long COVID complications include (but are not limited to) cardiorespiratory, gastrointestinal, endocrine, renal, neurological, and vascular disorders, in addition to fatigue, anxiety, and depression [6]. Pathogenic mechanisms have been hypothesised, with a recent large-scale cohort study demonstrating the detrimental impact of persistent inflammation on long-term secondary outcomes, based on the assessment of 368 plasma proteins [7]. Testing for long COVID remains insufficient; however, several diagnostic approaches are in development. Of relevance to potential laboratory tests, early research into biomarkers suggests that levels of extracellular vesicles and/or immune markers indicating high cytotoxicity could be indicative of long COVID [6].

There remains a paucity of data on the long-term impact of SARS-CoV-2 infection in relation to dysregulated T cell responses. Here, we use a flow cytometry method to explore T cell responses, specifically T cell receptor repertoire changes over a 12-month period in COVID-19 patients. We have evaluated patients at multiple timepoints across acute and convalescent phases of infection. We have included patient subgroups to capture disease severity (mild/asymptomatic, moderate, severe, and critical) alongside unvaccinated and vaccinated control participants without prior exposure to SARS-CoV-2. Studies investigating the differences between SARS-CoV-2 unvaccinated and vaccinated individuals [8] and individuals with a moderate, severe, or critical classification of COVID-19 disease [9] have been conducted; however, these do not assess whether there are any long-term immunological effects on the TCR repertoire following exposure.

The complexity of the TCR system is vast, with continually evolving hypervariability to mount an appropriate immune response to a diverse range of pathogens and cancers encountered throughout our lifetime. We point the reader towards the recent review by Watkins and Miles for a comprehensive appraisal of the genetics and architecture of the human TCR role in health and disease [10]. TCR repertoire diversity has been previously investigated in relation to exposure from a number of different pathogens, including Mycobacterium tuberculosis [11], Epstein–Barr Virus [12], and Cytomegalovirus [13]. More recently, in the wake of the COVID-19 pandemic, the assessment of the TCR repertoire has become a popular investigation for evaluating whether any observations or clinical predictions can be seen upon exposure to SARS-CoV-2. Previous studies have reported a decrease in the diversity of TCR clonotypes in COVID-19 patients when compared to healthy controls [9], and this has been associated with more severe disease [14,15]. These findings suggest that the success of the immune response to SARS-CoV-2, and ultimately, disease severity, is determined by TCR repertoire diversity. Effective immunity against a wide array of pathogens has been reported to be critically reliant on a broad TCR repertoire [13,16]. Poorer outcomes associated with a constricted TCR repertoire have been observed in other disease types, including HIV progression to AIDS [17] and metastatic breast cancer patients [18]. Limited TCR repertoire diversity, which is often observed in the elderly, has also been shown to correlate with poorer vaccination responses [19].

There are several techniques currently in use for the study of TCR repertoire composition, including PCR-based methods [20]. These methods can be time consuming and labour intensive, making them not suitable for a high throughput of samples. A more recent alternative method often used for TCR repertoire analysis is flow cytometry. The analysis of TCR repertoire specificities via flow cytometry offers a quick method which can measure the expression of T cell subsets on a single cell basis without requiring cell-sorting. The recent literature has described the application of machine learning to differentiate disease groups by their TCR repertoire signature [21], offering a novel and effective technique for analysing TCR repertoire data. The objective of this study was to assess for variance in the TCR vb repertoires of patient groups with different disease severities, and to test whether the TCR repertoire signatures of individual patients could be used independently to classify patients into disease severity groups. In this study, we use flow cytometry and machine learning techniques to investigate the outcome of TCR repertoire expression in a variety of infection groups, defined by their severity of COVID-19 disease. We demonstrate significant changes in the CD4+ T cell population size of specific TCR vb families in COVID-19 disease patients over a 12-month period, and between disease severity subgroups. Furthermore, we present a novel high-throughput platform for the classification of COVID-19 patients with different disease severities. Using flow cytometric TCR vb family data alongside backpropagation artificial neural networks, we were able to demonstrate outstanding discrimination for the classification of moderate and critical disease IPs, and excellent discrimination for severe disease IPs. These findings contribute new scientific knowledge to the immunological changes occurring at the T cell receptor level between disease severity groups and over time following infection with SARS-CoV-2.

## 2. Materials and Methodologies

### 2.1. Participants and Sample Collection

EDTA peripheral blood was collected from volunteers registered to the ‘Exploring COVID-19 specific immune responses in acute and convalescent phases of infection (EXCOVIR)’ clinical trial. This trial was undertaken in accordance with the Health Research Authority (IRAS283457). The patient groups included within this paper were the following: unvaccinated healthy (HC-UNVAC), vaccinated healthy (HC-VAC), mild–asymptomatic convalescent (MA), and in-patient (IP). The IP groups were further subdivided by severity of COVID-19 according to the World Health Organisation interim guidance [22] into moderate, severe, or critical disease categorisations. The HC-UNVAC cohort were recruited from 21 December 2020 until 15 February 2021. The HC-VAC cohort were recruited from 15 March 2021 to 11 May 2021. The MA cohort were recruited from 18 January 2021 to 20 January 2022. The IP cohort were recruited from 10 December 2020 to 10 November 2021. Sampling was conducted at acute (day 7) and convalescence (day 28, 6 months, 12 months) timepoints for IPs; at convalescence timepoints, only for MA; and at a single (recruitment) timepoint for HCs. Relevant individual patient demographics (age, sex, ethnicity, BMI, comorbidities, Glasgow coma score (GCS), and COVID-19 disease severity) are described in Supplemental Table S1. The HC groups (UNVAC and VAC) and the MA cohort were age matched; the IP severity groups (moderate, severe, critical) were age matched. The age matching of IPs with the HC and MA groups was not possible within this study; increased age is a known risk factor for hospitalisation following SARS-CoV-2; therefore, this was an accepted limitation. Moreover, as patient recruitment was performed during periods of national lockdown, HCs and MAs were recruited healthcare workers. For each timepoint, ANOVA testing was performed on laboratory results between each patient group (*p* < 0.05 was considered statistically significant at a 95% confidence level). We acknowledge that there will be differences between timepoints and severity groups in line with the recognised haematological features of COVID-19 reported in the literature [23].

SARS-CoV-2 seropositivity was determined by the Elecsys Anti-SARS-CoV-2 double-antigen sandwich assay format on the Roche Cobas 8000 platform. Both the spike and nucleocapsid variations of this assay were conducted, thus aiding the characterisation of unvaccinated and vaccinated healthy controls. Any patients recruited to the HC-UNVAC cohort that were shown to have nucleocapsid and/or spike antibody positivity were excluded from the cohort. Any patients recruited to the HC-VAC cohort who were positive for both spike and nucleocapsid antibodies were excluded, as they were likely to have been infected with SARS-CoV-2 previously.

### 2.2. TCR vb Analysis by Flow Cytometry

An analysis of 24 TCR Vβ was conducted using the IOTEST Beta Mark TCR Vβ repertoire kit as per the manufacturer’s recommendations (Beckman Coulter Inc., Brea, CA, USA). The kit is composed of 8 vials containing mixtures of conjugated TCR VB antibodies corresponding to 24 different specificities (Supplemental Table S2). The staining of peripheral blood samples was conducted using differing combinations of three TCR Vβ monoclonal antibodies, with each antibody being conjugated to either FITC, PE, or FITC/PE. A third colour T cell marker CD4 PC-5 was used to gate the specific cellular population. The samples were analysed using the Navios flow cytometer.

### 2.3. Statistical Analyses

Statistical analysis was conducted where sufficient data were collected. ANOVA analysis was conducted using GraphPad Prism 9.0 (GraphPad, Boston, MA, USA) software and MATLAB R2014b (MathWorks, Inc., Natick, MA, USA). Means, standard deviations, and *p*-values were calculated for each TCR vb family to identify differences between the investigated cohorts in the univariate analyses. For one-way ANOVA testing, *p* < 0.05 was considered statistically significant. Multivariate analyses were performed by means of principal component analysis (PCA) and backpropagation artificial neural networks (ANNs) within the MATLAB environment using the PLS Toolbox version 7.9.3 (Eigenvector Research, Inc., Manson, WA, USA) and the Classification Toolbox by Ballabio and Consonni [24].

PCA is a multivariate analysis technique employed for exploratory analysis and feature selection, where the original dataset (e.g., vb% expression for all the TCR vb families) is reduced to a fewer set of variables called principal components (PCs), which account for most of the original variance in the dataset [25]. These components are orthogonal to each other, and they are built in a decreasing order of explained variance, where the first PC accounts for the most variance in the dataset, following by the second PC, and so on. Each PC is composed of scores and loadings, the first representing the variance on the sample space direction and the latter on the original variable direction. Therefore, the scores allow one to assess the similarities or dissimilarities between the observed samples (patient cohorts) through visual patterns, while the loadings provide information of the weight of each variable (TCR vb family) in the pattern observed. Cluster analysis on PCA score plots was used to demonstrate discrimination between groups. A 95% confidence ellipse was added to illustrate possible ‘outliers’, that is, any patients falling within 5% of the data variance. It should be noted that any patients falling outside the 95% confidence ellipse are not necessarily statistical outliers; rather, it can be assumed that they contain features that are different from the other patients within the group.

Backpropagation ANN is a deep learning algorithm capable of predicting highly complex observations through a learning process based on the Deepest-Descent technique [26]. An ANN was employed to classify mild–asymptomatic convalescents and the IP groups into pre-defined categories (moderate, severe, or critical) based on their TCR vb family CD4+ percentage. So, given the cellular response, the algorithm is capable of assigning a specific patient to one of these three groups. The ANN model was built using around 70% of samples for training and 30% for testing. The training and test sets were selected based on the Kennard–Stone (KS) uniform sampling algorithm [27]. The optimisation of ANN training parameters was made by venetian blind cross-validation with five data splits. Only the TCR vb families with *p*-values bellow 0.05 observed in the univariate analyses between the groups (MA, IPs moderate, severe, and critical) were used as input data for the ANN model. The data were autoscaled before multivariate analysis by PCA and ANN was performed.

## 3. Results

We examined the CD4+ percentage differences in 24 TCR vb specificities using a flow cytometry method (Beta Mark; Beckman Coulter). Multivariate analysis methods (principal component analysis (PCA) and backpropagation artificial neural networks (ANNs)) were used to compare patient subgroups and timepoints in a series of experiments, as described in Table 1. Following these nine comparator analyses (described in Table 1), TCR vb families with significant variances between subgroups were identified. In total, sixteen specific TCR vb family specificities demonstrated significance over the different experiments, with seven families identified as significant in four or more of the nine individual experimental comparisons. In order of highest frequency is as follows: TCR vb 20 (significance in 6/9), vb 5.2 (significance in 5/9), vb 4, vb 12, 13.1, vb 17, and vb 18 (significance in 4/9). These findings suggest a role of restricted TCR vb family specificities in relation to the disease severity and time-course of disease in COVID-19. The impact of individual TCR vb family differences has been further explored in relation to specific disease severity subgroups and timepoints within this study.

Two classification models were built for the prediction and segregation of patient subgroups based on severity of disease. Of highest performance (accuracy 94%), a backpropagation ANN model enabled the successful classification of COVID-19 IPs into moderate, severe, or critical disease. Excellent AUC scores for ROC analysis (0.88 for severe, and 0.94 for moderate and critical patients) were achieved, highlighting the utility of TCR vb family analysis as a future predictive tool. The inclusion of MA patients into the backpropagation ANN model enabled the good classification of patient subgroups (accuracy 84%), showing the highest performance when separating MA patients from IPs in the critical disease category. The results from each patient group regarding leukocyte count, lymphocyte count, CD4+ T cell count, CD8+ T cell count, and CRP can be seen in Table 2. Of note, significant differences between patient groups were determined for CD4+ T cell count and CRP level at the 6-month timepoint and CRP level at the 12-month timepoint.

### 3.1. Significant Variance in Six TCR vb Families Following ANOVA Testing Between All Nine Patient Groups, Minor Clustering of Day 7 IP Samples from Remainder of Other Study Populations

**Figure 1:**
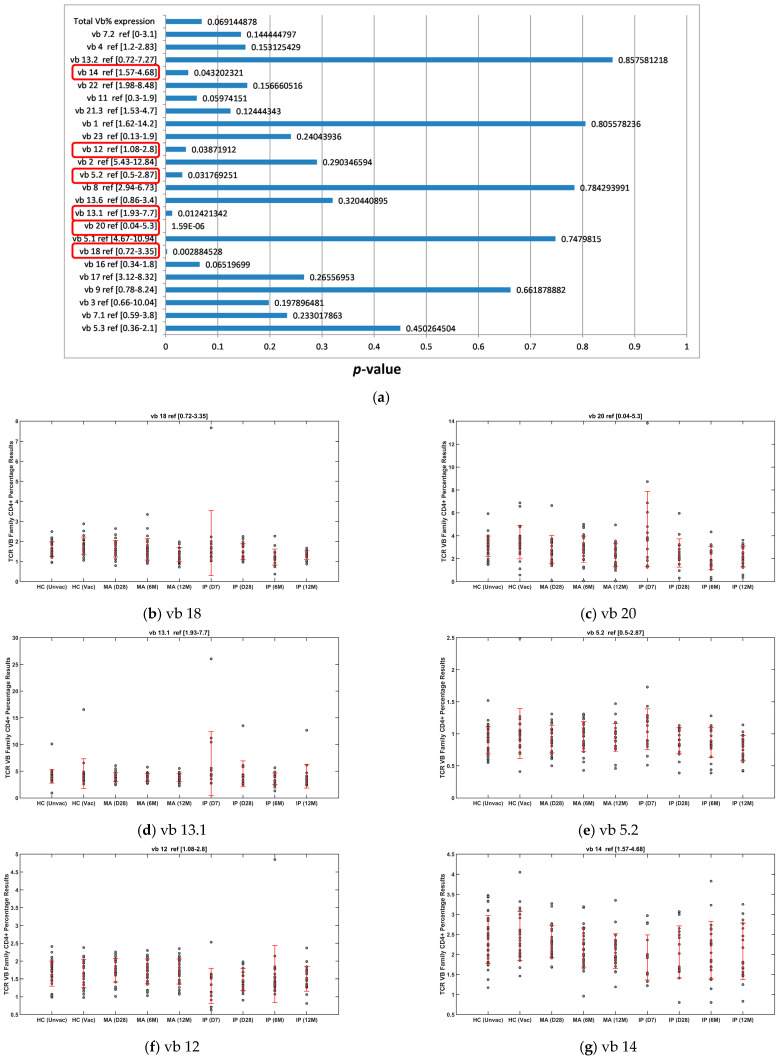

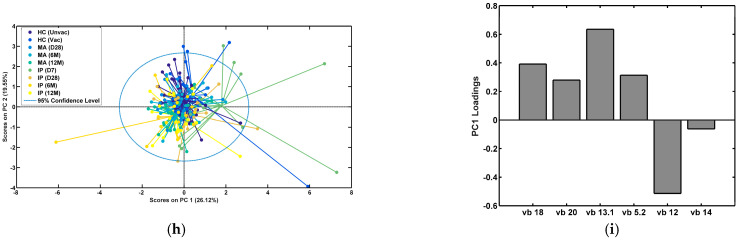
Exploratory analysis comparing 9 major subgroups. (**a**) ANOVA test for each TCR vb family (*p* < 0.05 is considered statistically significant [circled]). (**b**–**g**) Mean CD4% and SD for six vb families demonstrating significant differences by ANOVA. (**h**) Exploratory analysis by PCA using the TCR vb families with *p* < 0.05. Some clustering for IP day 7 (D7) distances the group from others. (**i**) Key loadings on PC1 responsible for distinguishing IP (D7) group.

We performed exploratory analysis for the complete dataset to assess for significant variances in CD4 TCR vb family percentages between all cohorts (HC, MA, and IP). The six families demonstrating significant variance across the complete dataset were vb 14, vb 12, vb 5.2, vb 13.1, vb 20, and vb 18. The CD4+ percentage mean and standard deviation results for each vb family illustrate that the major differences relate to the IP cohort on day 7 (Figure 1b–g). For TCR vb families vb 18, vb 20, vb 13.1, and vb 5.2 (Figure 1b–d, respectively), an increased mean percentage of CD4+ T cells can be observed for IP D7 samples compared to all other cohorts and timepoints. For TCR vb 12 and vb 14 (Figure 1f and 1g, respectively), these families demonstrate a reduction in CD4 T cell percentages in the IP D7 samples compared to all other cohorts and timepoints. Of interest, for TCR vb 14, there was a reducing trend in the percentage of CD4 T cells in MA patients from day 28, to 6 months to 12 months, in contrast to the IP patients, in which CD4 T cell percentages trend upwards from day 7, to day 28, to 6 months. At the 12-month timepoint, both the IP and MA cohorts illustrate comparable percentages of CD4 T cells for the TCR vb 14 family. This finding is confirmed by PCA, performed using only the vb families identified as being significantly different by ANOVA testing. Minor clustering for the IP day 7 samples can be seen to distance this group from the remaining cohorts (Figure 1h). No further separation between the other groups can be observed. The examination of the loadings on PC1 responsible for distinguishing the IP (D7) group demonstrates that the main variables with positive weights are vb 13.1, vb 18, vb 5.2, and vb 13.1. Variables vb 12 and vb 14 have negative weights (Figure 1i).

### 3.2. Significant Alterations in TCR vb Family Expression Supports Clustering Separation Between Disease Severity Cohorts MA Patients and IPs (Moderate, Severe, Critical)

**Figure 2:**
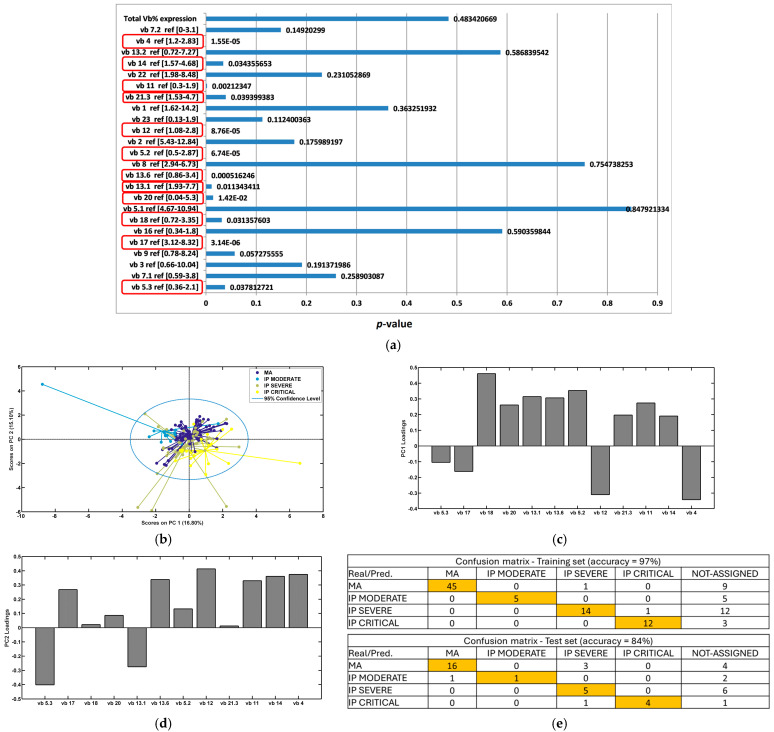
Exploratory analysis by PCA comparing COVID-19 patients (all severity groups; inclusive of all collected timepoints). (**a**) ANOVA test for each TCR vb family (*p* < 0.05 is considered statistically significant [circled]). (**b**) PCA performed using the TCR vb families with *p* < 0.05. Clustering separation between the groups can be observed. (**c**) Key loadings (features) on PC1. (**d**) Key loadings on PC2. (**e**) Classification modelling using backpropagation artificial neural networks (ANNs); the model shows good separation for MA and IP critical, and lower performance is noted for IP severe followed by IP moderate (correct responses shaded in yellow).

Significant variance in CD4 TCR vb family percentages between disease severity cohorts (MA, IP moderate, IP severe, and IP critical) were determined by ANOVA testing (Figure 2a). In this analysis, TCR vb data were analysed from MA patients (day 28, 6 months, 12 months), and IP data were taken from all timepoints analysed (day 7, day 28, 6 months, and 12 months). Significant variance (*p* < 0.05) was identified for 12 of the 24 TCR vb families. The families demonstrating significant differences between the cohorts were vb 14, vb 12, vb 5.2, vb 13.1, vb 18, and vb 20 (as observed previously when comparing all groups), in addition to families vb 13.6, vb 4, vb 11, vb 21.3, vb 17, and vb 5.3. In this analysis, the timepoint data were grouped together for each disease severity group.

Clustering separation between the groups was observed using PCA for the 12 significant TCR vb families, as illustrated in Figure 2b. Positive and negative loadings on PC1 (Figure 2c) and PC2 (Figure 2d) identify the key TCR vb families responsible for distinguishing between the disease severity groups. Using the TCR vb analysis data from all timepoints, a classification model was built using backpropagation artificial neural networks (ANNs). The model had high accuracy (84%) and the greatest performance observed for classification of MA patients and IP critical patients. The classification of IP moderate patients showed the lowest performance with the ANN model, followed by IP severe patients (Figure 2e). These findings suggest that given the TCR vb repertoire of an individual, the model can successfully segregate patients into disease severity groups from day 7 for IPs and day 28 for MA patients.

### 3.3. Significant Alterations in TCR vb Family Expression Demonstrates Clustering Separation Between Disease Severity Cohorts (Inclusive of Data at All Timepoints)

**Figure 3:**
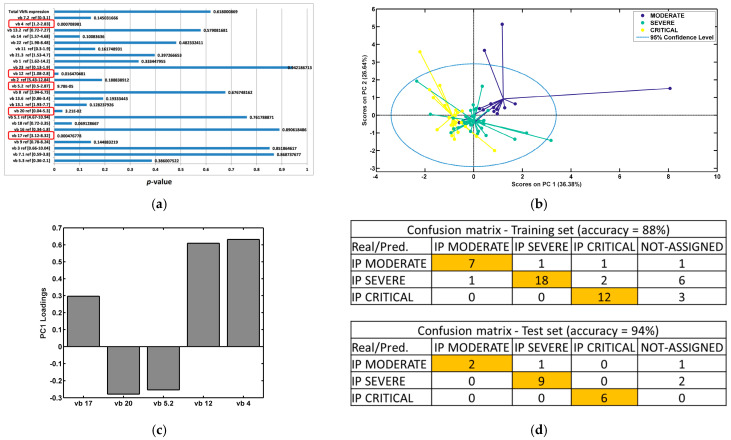

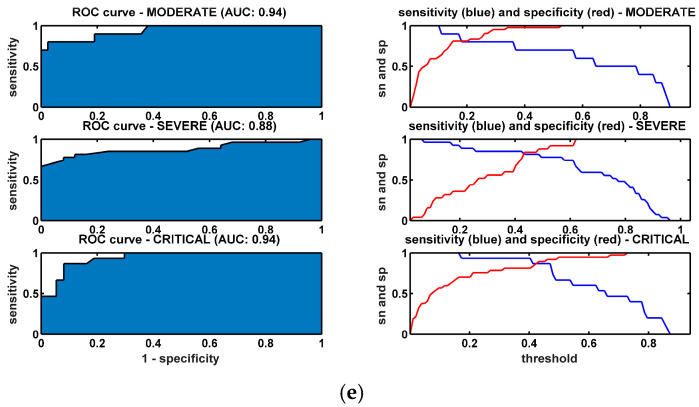
Exploratory analysis by PCA comparing COVID-19 IP severity groups (all timepoints). (**a**) ANOVA test for each TCR vb family (*p* < 0.05 is considered statistically significant [circled]). (**b**) PCA performed using the TCR vb families with *p* < 0.05. Good clustering separation between the groups can be observed. (**c**) Key loadings (features) on PC1. (**d**) Classification modelling using backpropagation artificial neural networks (ANNs); good classification between IP severity groups can be obtained (correct responses shaded in yellow), thus, given the TCR VB family CD4+ percentage, the model can predict if the patient is moderate, severe, or critical. (**e**) ROC analysis curves; performance characteristics indicate outstanding discrimination for classification of moderate and critical disease IPs, and excellent discrimination for severe disease IPs, sensitivity (blue), specificity (red).

Analysis of disease severity cohorts (moderate, severe, and critical) revealed significant alterations in TCR vb family expression, illustrated through ANOVA (Figure 3a) and good clustering separation (Figure 3b). TCR vb data were analysed from IPs inclusive of all timepoints (day 7, day 28, 6 months, and 12 months). Significant variance (*p* < 0.05) was identified for five of the 24 TCR vb families. Positive and negative loadings on PC1 identified the key TCR vb families responsible for distinguishing between the disease severity groups (Figure 3c). Classification using backpropagation ANN demonstrated excellent accuracy (94%); thus, for an unknown patient, the model could predict with excellent accuracy whether the patient was a moderate-, severe- or critical-disease patient based on TCR vb family data (Figure 3d). Receiver operator curve (ROC) performance characteristics were calculated for each disease severity cohort. Area under the curve (AUC) scores demonstrated excellent discrimination for severe IPs (0.88) and outstanding discrimination between moderate and critical IPs (both 0.94). Further, these findings suggest that the severity of infection has a significant and lasting impact on the TCR vb repertoire, given that the classification model successfully segregates patients into disease severity groups regardless of timepoint consideration.

### 3.4. Significant Alterations in TCR vb Family Expression During Acute COVID-19 Disease and Convalescence Period

**Figure 4:**
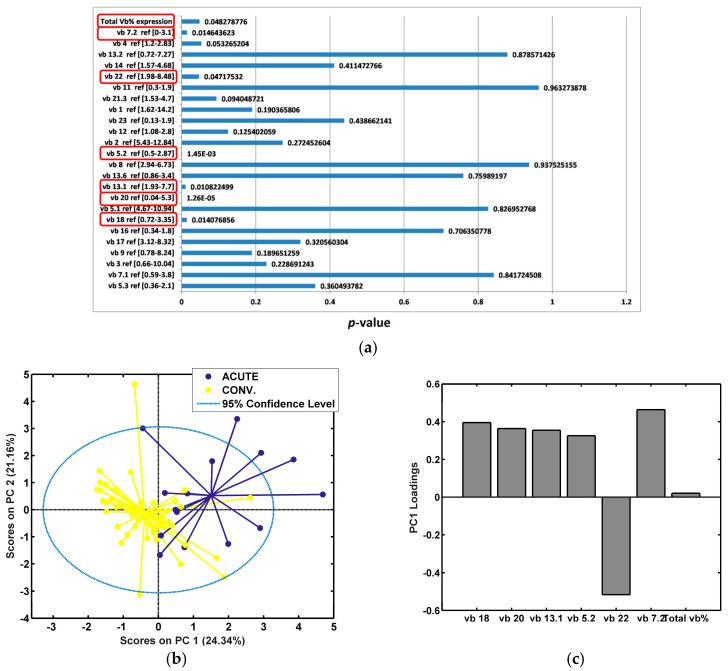
Exploratory analysis by PCA comparing IPs during acute infection (day 7) or convalescence (day 28, 6 M, 12 M). (**a**) ANOVA test for each TCR vb family (*p* < 0.05 is considered statistically significant [circled]). (**b**) PCA performed using the TCR vb families with *p* < 0.05. Clustering separation between the acute and convalescent group can be observed. (**c**) Key loadings (features) on PC1.

To assess TCR vb family changes during acute infection (day 7) compared to the convalescent period (day 28, 6 months, 12 months), IP TCR vb family variances were examined by ANOVA (Figure 4a). Disease severity was not considered during this analysis. We identified six significant differences between TCR vb family expression separating patients in acute infection from those in convalescence (TCR vb 7.2, vb 22, vb 5.2, vb 13.1, vb 20, vb 18). Of particular interest, this was the sole analysis experiment in which the total Vb% expression was significantly different between the two groups, acute vs. convalescent.

**Figure 5:**
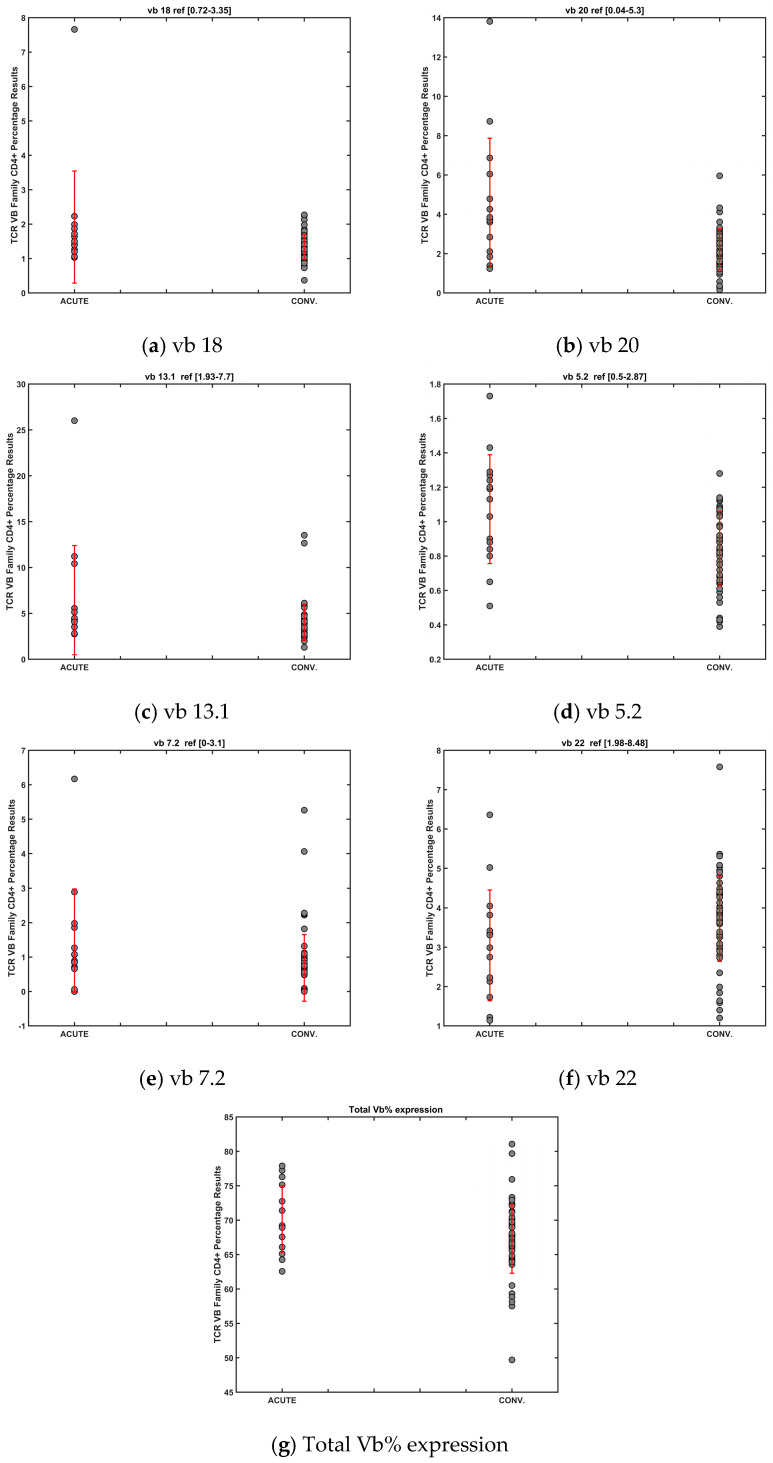
Mean CD4+ percentage and SD for 7 vb families and total vb% expression, demonstrating significant differences between acute and convalescent patients by ANOVA testing.

PCA cluster analysis demonstrated good separation between the two groups (Figure 4b). Of note, TCR vb 7.2 and vb 22 were identified as unique variances to this analysis, whereas the other families have shown significant differences in the previous exploratory analysis. This may suggest that these two families play key roles in the different stages of infection. In support of this, PCA revealed TCR vb 7.2 to have the highest positive loading, important for the separation of acute patients. TCR vb 22 had the largest negative loading, important for the separation of convalescent patients (Figure 4c). To further examine differences between acute and convalescent timepoint data, mean CD4+ percentages were calculated for each significant TCR vb family (Figure 5). As noted earlier, relatively high SDs limited trend interpretation; however, for five of the six TCR vb families (vb 7.2, vb 5.2, vb 13.1, vb 20, vb 18), the mean CD4+ percentages trended downwards from acute to convalescence. The percentages of CD4+ cells specific to TCR vb 22 were those of the only family expanded in the convalescent patients compared to acute patients. The significant difference observed in total Vb% expression was of particular interest. The analysis of TCR vb families in this study covers 24 different families (approximately 70% coverage of a normal TCR Vb repertoire); the higher percentage of total vb expression reported indicates a less diverse TCR repertoire [28]. Our results illustrate that total vb expression decreases in convalescence, thus TCR vb diversity is greater in convalescence than during acute infection. Whilst we cannot attribute cause and effect to the trends observed across these specific TCR vb families, the expansion and contraction of these populations may provide greater insight into TCR repertoire diversity changes over time and the overall dynamics of immune response to SARS-CoV-2.

Individual timepoint analysis (day 7, day 28, 6 months, and 12 months) by PCA for IPs showed no separation between the convalescent timepoints; there was separation from the acute patients (day 7) as previously illustrated, with TCR vb 5.2 and vb 20 identified as the PCA loadings responsible for the separation (Supplemental Figure S1).

**Figure 6:**
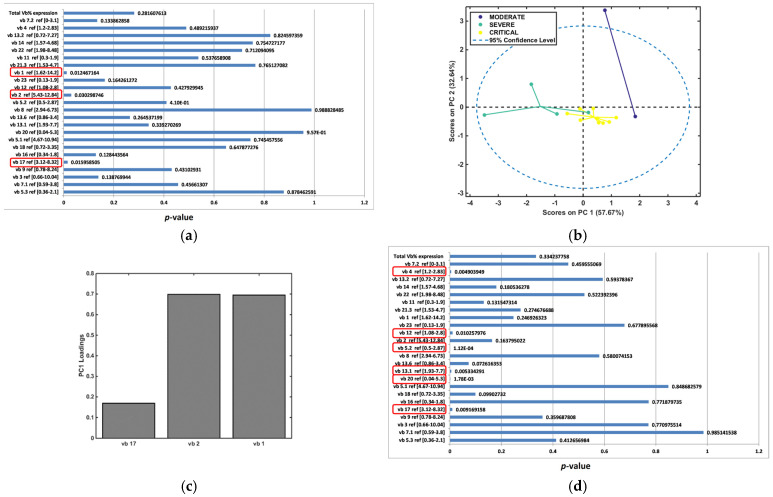

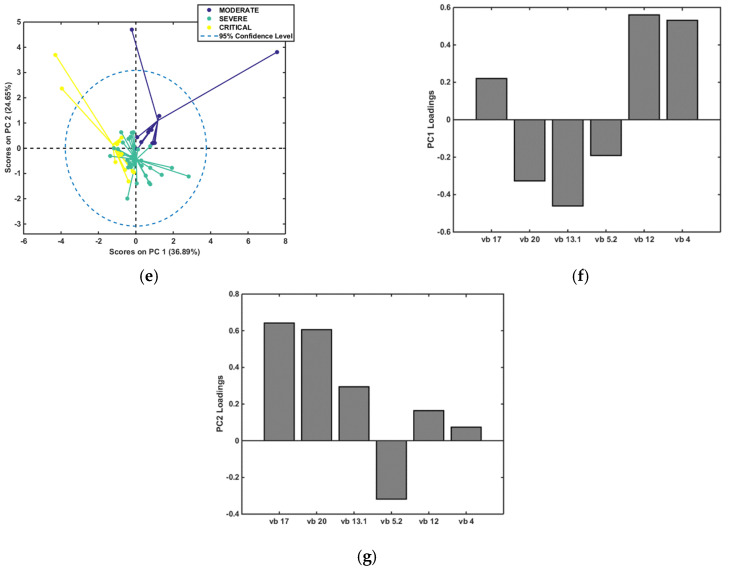
Exploratory analysis by PCA comparing COVID-19 IP severity groups. (**a**) ANOVA test for each TCR vb family (*p* < 0.05 is considered statistically significant [circled]) during acute infection period. (**b**) PCA using TCR vb families with *p* < 0.05—better clustering separation is observed along PC1 (horizontally). (**c**) Key loadings (features) on PC1. (**d**) ANOVA test for each TCR vb family (*p* < 0.05 is considered statistically significant [circled]) during convalescent infection period. (**e**) PCA using TCR vb families with *p* < 0.05—good clustering separation is observed along PC1 (horizontally) and PC2 (vertically). (**f**) Key loadings on PC1 important for moderate and critical separation. (**g**) Key loadings on PC2 important for moderate and severe separation.

The ANOVA analysis of IP severity groups during acute infection (day 7) showed significant variance between three TCR vb families (TCR vb 1, vb 2 and vb 17) (Figure 6a). PCA demonstrated a clustering of patient severity groups, with better separation on PC1 (Figure 6b). TCR vb 2 and vb 1 had the highest positive loadings on PC1, followed by vb 17, which is an important feature for the segregation of moderate disease severity patients (Figure 6c). As sampling for TCR vb analysis was only conducted on day 7 of acute disease, the sample size within each severity group was small, limiting the strength of these findings. Convalescence period sampling occurred at three timepoints, day 28, 6 months, and 12 months, resulting in larger sample sizes for each disease severity group. ANOVA revealed six significant TCR vb family differences between severity groups (TCR vb 4, vb 5.2, vb 12, vb 13.1, vb 17, and vb 20) (Figure 6d). Good PCA clustering separation was observed on PC1 and PC2, with moderate-severity patients clustering more discretely from the severe and critical patients (Figure 6e). Positive loadings on PC1 (Figure 6f) and PC2 (Figure 6f) were important for discriminating moderate patients, whereas negative loadings were important for severe and critical disease severity.

**Figure 7:**
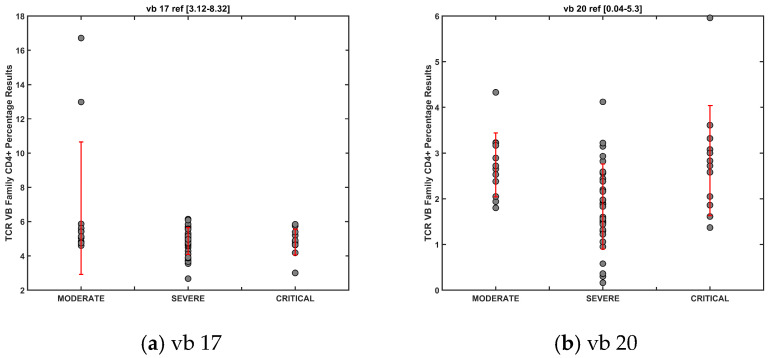

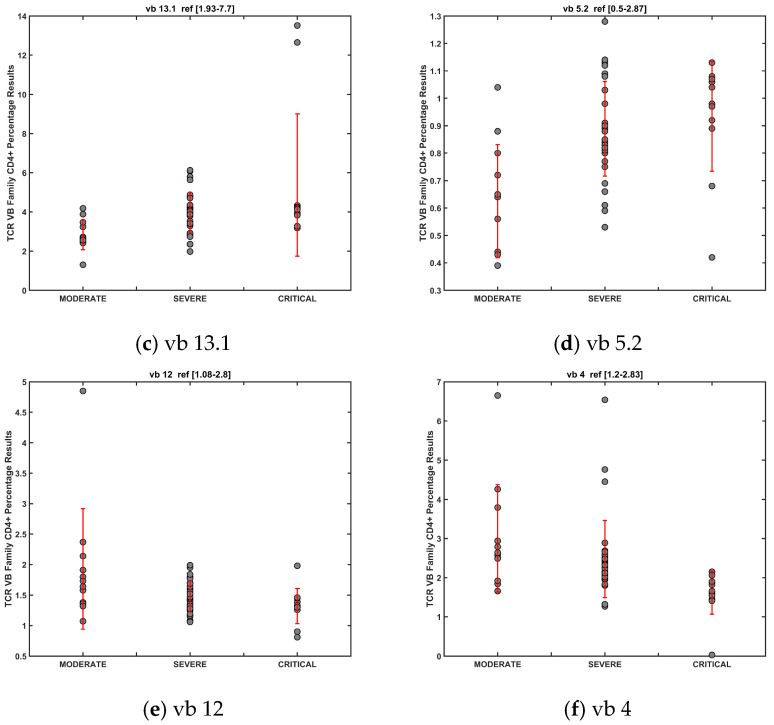
Mean and SD for 6 vb families demonstrating significant differences by ANOVA severity group convalescent IPs.

Mean CD4+ populations decreased with increasing disease severity for TCR vb 17, vb 12, and vb 4, whilst expanding for vb 13.1 and vb 5.2. For TCR vb 20, patients with moderate or severe disease were found to have larger CD4+ populations than patients with severe disease (Figure 7). Higher SDs due to within-group variability is a limitation of this trend analysis; however, our earlier ANN classification model data support the finding that specific TCR vb repertoire changes can be observed and used to classify disease severity IP groups.

### 3.5. TCR vb Repertoire Is Not Significantly Modified Following Vaccination in Healthy Controls

**Figure 8:**
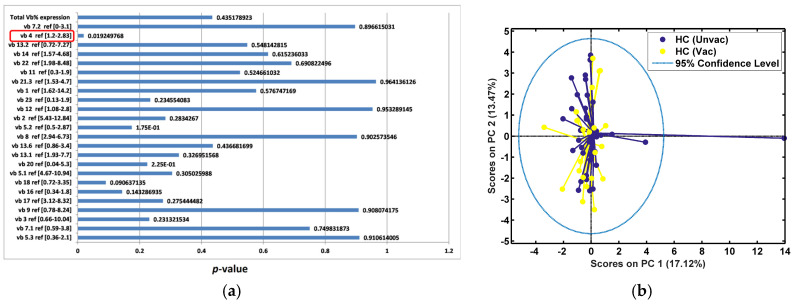

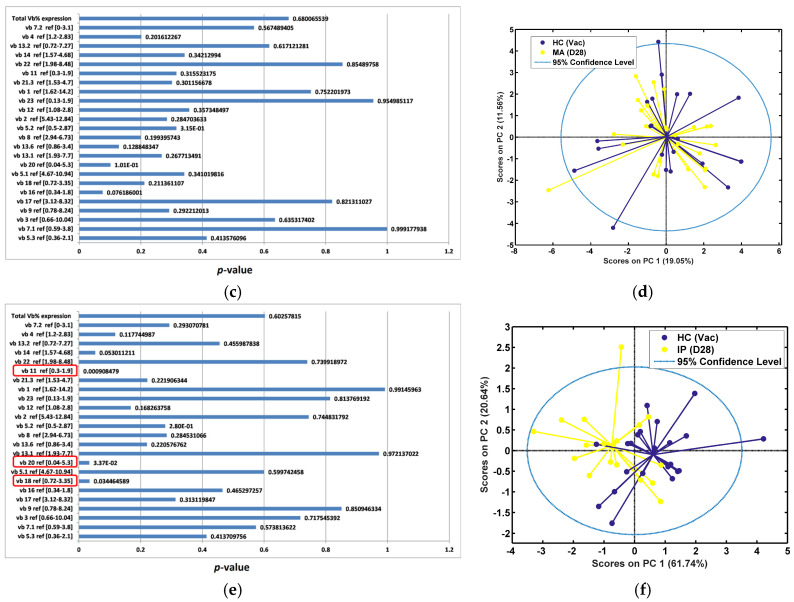
Exploratory analysis by PCA comparing vb family expression in HC-VAC with HC-UNVAC (**a**,**b**); mild/asymptomatic (**c**,**d**); and IPs at day 28 post infection. (**e**,**f**). (**a**,**c**,**e**) ANOVA test for each TCR vb family (*p* < 0.05 is considered statistically significant [circled if present]). (**b**,**d**) PCA using all TCR vb families, poor separation observed. (**f**) PCA performed using TCR vb families with *p* < 0.05. Some clustering separation between HC-VAC and IP group can be observed. (**e**) Key loadings (features) on PC1.

In our final analyses, we examined the HC groups in greater detail. We assessed TCR vb family changes, comparing HC-VAC and HC-UNVAC participants (Figure 8). ANOVA demonstrated significant variance in a single family, TCR vb 4, between the two groups (Figure 8a). It is not possible to perform PCA using a single feature, thus PCA was performed using all vb families. We demonstrated poor separation between the groups and overlapping clusters (Figure 8b). This suggests that CD4 TCR vb family expression is not significantly modified following vaccination.

### 3.6. TCR vb Family Changes Are Observed at Day 28 in IPs but Not Mild/Asymptomatic Patients When Compared to Healthy Vaccinated Controls

TCR vb family differences between vaccinated HCs and COVID-19 disease patients were compared for both MA and IPs (all severity groups) at day 28 (Figure 8c–f). ANOVA testing did not reveal any significant difference for any of the 24 TCR vb families when comparing HC-VAC participants to MA (Figure 8c). PCA confirmed this finding with the complete overlapping of clusters (Figure 8d). In contrast, the comparison of the HC-VAC cohort with IPs revealed three TCR vb families with significant variance between the two groups (TCR vb 18, vb 20, vb 11) (Figure 8e). PCA performed using these features demonstrated discrete clustering and separation between the two groups on PC1 (Figure 8f). All three families had positive loadings on PC1 and notable features for the segregation of the HC-VAC group.

## 4. Discussion

Bias in the T cell repertoire has been widely reported among pathogenic diseases, having clinical consequences in both disease and vaccination responses [20]. Following three seminal papers in the early 1990s [29,30,31], numerous reports of TCR bias in infectious disease have been published. Most of the work has focussed on the herpesviruses CMV and EBV; however, TCR bias has also been noted in other human pathogens [32,33,34,35,36,37,38,39,40]. Of relevance, several of these observations have been made in infections with RNA viruses, which possess extraordinary mutagenic and replicative potential.

Here, we report a novel application using machine learning and the flow cytometric analysis of the TCR-Vβ repertoire to assess COVID-19-associated repertoire changes over a 12-month period. Flow cytometry is widely used to investigate the human TCR repertoire, as it quickly provides insight into the TCR repertoire of many different T cell subsets without the need for laborious cell sorting. Whilst this method is popular due its speed, relative ease, and quantitative features, the data are constrained by the lack information on the CDR3 residue composition, being of low resolution, and having incomplete coverage (only 24 vb-specific antibodies are commercially available). At present, due to the staggering hypervariability potential within the TCR repertoire, no single platform has proven to be completely capable in the assessment of true TCR diversity. Nevertheless, the flow cytometric approach provides a good illustration, within a single day, as to whether the repertoire of a T cell population is diverse or biassed.

We illustrate significant TCR vb repertoire changes associated with COVID-19 infection over a 12-month period. Through undertaking multiple experiments, we were able to determine changes in the TCR vb repertoire between different disease severity subgroups, and at different timepoints, in addition to healthy non-vaccinated and vaccinated controls. The significant TCR vb families found to have highest frequency across the different experiments are recorded in Table 3, with TCR vb 20 appearing most often as a discriminating feature across six of the nine experiments. Thereafter, TCR vb 5.2 was identified in five experiments; TCR vb 4, vb 12, and vb 17 in four experiments, and families appearing three times or fewer, including TCR vb 18, vb 13.1, vb 14, vb 11, vb 22, vb 13.6, vb 7.2, vb 2, and vb 1. The TCR vb families demonstrating significant variances across multiple experiments may suggest a direct role in the immune response to SARS-CoV-2.

Of greatest clinical importance, we demonstrate the successful application of a machine learning method, backpropagation artificial neural networks, to separate subgroups based on disease severity for IPs (moderate, severe, or critical). Our identification of a TCR vb signature associated with disease severity serves as a first step to support patient triage and subsequent patient management for optimal care. The goal of ANNs is to build a predictive model whereby the computer “learns” how to identify patient severity based on immunological response parameters. Applied clinically, it would be possible to know in advance what the disease severity will be. Whilst some of the data used to train the model were collected after the clinical event (patient hospitalisation), once trained, the model was able to predict severity blindly based on the TCR vb input. We demonstrated outstanding discrimination between moderate, severe, and critical patients, with ROC analysis resulting in AUC values of 0.94, 0.88, and 0.94, respectively (Figure 2). The TCR vb signature for this analysis included five significant TCR vb families; vb 20 and vb 5.2 percentages trended upwards when comparing patients with moderate disease to critical disease, whereas vb 4, vb 12, and vb 17 trended downwards. The classification model for IPs includes TCR vb data collected at all timepoints (day 7, day 28, 6 months, 12 months), indicating that the observed signature persists over time. It has been reported that the diversity and clonality of the TCR repertoire peaks within 8–14 days of SARS-CoV-2 infection, then contracts slightly [41,42] before returning to basal levels one week after viral clearance [43]. Our findings are in line with other studies, showing thatSARS-CoV-2-specific TCR repertoire changes can be detected up to 15 months after virus elimination, with reports showing a slight decrease [4,41,44] or increase in clonal diversity [45].

We observed higher percentages of TCR vb 20 and vb 5.2 in critical disease patients; this may reflect a reduced overall TCR diversity and a risk of more severe disease. A limited TCR diversity has been reported in patients with severe COVID-19 disease [14,15], presumably because SARS-CoV-2-specific clones continue to expand if infection persists in hospitalised patients [44]. As all timepoints were included and grouped together, it was not possible to determine whether SARS-CoV-2 infection skewed the TCR repertoire in the critical patients, or whether other factors, such as previous infectious or other pathogenic disease, had already had an impact on TCR vb diversity [20]. Alternatively, lower TCR repertoire diversity pre-COVID-19 disease may be a prognostic factor, explaining the higher risk of severe disease and increased death rate in the elderly [1,2,46,47,48]. Ageing causes a decline in TCR diversity, negatively impacting the immune response to other pathogens, such as human influenza A virus; it is plausible that the same is true for the immune response to SARS-CoV-2 virus [49,50]. With regard to the 3 vb families (vb 4, vb 12, and vb 17) showing expansions in moderate disease compared to critical, we hypothesise that these families may be advantageous, proliferating early to aid in the elimination of the virus and preventing a more severe or critical disease course. Reports of cross-reactive and public clonotypes offering protection against SARS-CoV-2 have not been fully substantiated [51,52,53,54,55,56,57]; however, an early expansion of vb families specific to SARS-CoV-2 epitopes may offer protection against severe disease.

To examine the complete dataset in a single experiment, we included all nine participant subgroups. PCA cluster analysis was unable to show discrete clustering between subgroups with the exception of the IP day 7 subgroup. This suggests that the greatest changes in TCR vb repertoire diversity occurred in the acute stage of hospitalised COVID-19 patients, in line with findings reported in the literature [42,58]. Focussing on this subgroup, we noted clonal expansion in four of the six families (TCR vb 18, vb 20, vb 13.1, vb 4.2); this may suggest a reduced overall receptor repertoire diversity and thus a poorer prognostic outcome for these patients, as discussed earlier. For TCR vb 12 and vb 14, a reduced population was observed in the IP day 7 group; if, as previously postulated, these families offer a protective role, failure to expand in the early stages of disease may result in severe disease and thus hospitalisation. Whilst we cannot demonstrate cause and effect within this study, interestingly, the expansion of these families seems to be delayed in hospitalised patients. We subsequently observed the expansion of these families at the day-28 and 6-month sampling points, before levelling off 12 months post infection. These findings suggest a key role for these TCR vb families in the immune response to SARS-CoV-2 infection.

Further analysis of IP-only data illustrated clear separation between TCR vb repertoire signatures during acute infection and the convalescent period. Of significance, five families (vb 18, vb 20, vb 13.1, vb 5.2, and vb 7.2) were shown to be expanded in the acute period compared to in convalescence, whereas one family (vb 22) expanded in convalescence. The comparative expansion of five TCR vb families at day 7 may be an indication of patients with a reduced overall TCR vb diversity and increased severity of disease. We postulate that these changes may reflect dominant SARS-CoV-2-specific T cell clones, continuing to proliferate due to the persistence of the virus. Alternately, the expanded clones represent exhausted and/or anergic populations, or T cells which have been subject to immune evasion mechanisms by the virus, as has been previously reported for herpesviruses such as EBV and CMV [59,60]. A similar mechanism has been postulated in age-related immune deficiency, whereby the narrowing of virus-specific TCR repertoires and accumulation of CD28^negative^ effector/memory T cells occurs in elderly individuals, restricting the available space for functioning T cells [49,50,61]. Of interest, TCR vb 20 and vb 5.2 demonstrate the greatest expansions in the grouped IP data with the highest disease severity (IP critical); moreover, these families have been identified most frequently as significant within the nine analyses (six and five times, respectively). This may highlight a dominant role of these clones during active infection. We recognise that our data can only make an indirect case for cause and effect, as the patients with critical disease are likely to have had prolonged SARS-CoV-2 viremia and chronic inflammatory responses. We have demonstrated a skewing of the TCR vb family from the acute to convalescent period of infection, in line with reports for other viral infections [62,63]. We report that the majority TCR repertoire changes had occurred by day 28, in keeping with other TCR repertoire studies during SARS-CoV-2 infection;, with peak changes and reduced TCR repertoire diversity occurring between days 8 and 14, before returning to basal levels one week post viral elimination [4,41,42].

Between IP severity groups at acute (day 7) and grouped covalent timepoints, we observed cluster separation between the moderate, severe, and critical patients alongside significant vb family variances (Figure 6). As the number of data points was small, interpretation is limited; however, discrete separation was apparent on PCA scores plots, particularly along PC1. The relative differences observed in TCR vb families in convalescence may occur because of previously expanded clones contracting, supporting the development of a more diverse repertoire post infection. Support for this theory can be evidenced through studies in other viral infections, which demonstrate that highly diverse repertoires are shown to be protective in EBV, CMV, and HIV-1 [64,65]. It is likely that such repertoires represent high-avidity, high-affinity, and high-functionality T cells, which confer the greatest immune protection [66,67]. We observed patients with moderate disease severity clustered separately to patients with severe and critical disease, suggesting that the differences between TCR vb family expression appears to be of prognostic significance in COVID-19 disease. A protective role of public clonotypes in COVID-19 has previously been suggested, with some epitopes of SARS-CoV-2 giving rise to TCRs with shorter CDR3 regions, arising from V(D)J-rearrangement events that occur with greater probability [68]. Similar protective, pathogen-specific clonotypes have previously been suggested to play a crucial role in the control of other viruses, such as CMV, EBV, and Adenovirus [69,70].

In the final analyses, we explored TCR vb differences in HC groups and MA patients. We observed a complete lack of clustering discrimination between HC-VAC and HC-UNVAC groups and HC-VAC and MA patients. This suggests that the TCR vb family repertoire is not evidently skewed following vaccination. However, as the period post-vaccination to recruitment was not standardised (mean 87 days (60–140)), any acute repertoire changes immediately following vaccination may not have been detected. Conversely, when comparing the TCR vb family changes in HC-VAC vs. IPs, we observed three significant vb family population differences and discrete clustering by PCA (Figure 8f). Thus, a different response is evoked in patients with severe natural infection compared to vaccination responses or mild disease. We further hypothesise that the HC and MA groups may share protective immunological similarities, such as a highly diverse TCR vb repertoire, inclusive of public clonotypes with cross-reactivity to SARS-CoV-2 epitopes, as suggested by several groups in the literature [55,57,71,72]. One of the most cross-reactive SARS-CoV-2 nucleocapsid epitopes reported in unexposed HLA-B7+ individuals is the immunogenic N105–113 peptide SPRWYFYYL (SPR) [73,74,75]. It has been proposed that pre-existing immunity may have been generated by previous infection with coronaviruses, given the peptide sequence is identical in SARS-CoV-2 and SARS-CoV-1, and has only minor residue difference in four seasonal coronaviruses (OC43, 229E, NL63, and HKU-1) [76,77]. These cross-reactive progenitors arise from a highly diverse TCR repertoire, and are thought to offer increased protection to SARS-CoV-2 infection, as polyfunctional SPR-specific T cell responses with high avidity have been detected in patients with mild disease compared to those with severe disease [74].

## 5. Conclusions

Despite the fact that COVID-19 has now been moved into the category of seasonal respiratory infections, studies examining the adaptive immune response and variances between disease severity groups are still relevant and may help in the future to assess the complications of viral infections in the long term.

For COVID-19, the influence of comorbidities was recognised since the earliest days of the pandemic. However, establishing causality and underlying mechanisms continues to be a challenge due to wide patient variability and other confounding factors. Comorbidities associated with altered immune function, such as latent CMV, have been shown to be strongly associated with severe disease, independent of age and other conditions [78]. Taken together, the data from the analyses within this study support the hypothesis that a largely diverse and polyclonal repertoire is associated with health, whilst, in the context of SARS-CoV-2 infection, it becomes more restricted; this may be due to the virus-specific clonal expansion or deletion of T cells, and/or pre-existing factors. This is a small and preliminary study, using a novel analytical approach (flow cytometric analysis of TCR vb repertoire alongside machine learning) to classify COVID-19 IPs with differing disease severities. A larger dataset set is required to corroborate our findings and test predictive models. Further, in relation to specific TCR vb family clonal expansions, the impact of SARS-CoV-2 serotypes and variants of escape would be of interest to examine in a future study. We have identified key variances between patient subgroups over the acute and convalescent period of SARS-CoV-2 infection; however, the TCR vb family signatures identified in the current study do not provide proof of cause and effect. Rather, our findings provide new evidence towards understanding of the impact of SARS-CoV-2 infection on TCR repertoire diversity, including longitudinal changes over a 12-month period for patients with mild/asymptomatic, moderate, severe, and critical COVID-19 disease. Following the successful application of this technique in classifying patients into disease severity groups, the next steps will be to explore the future development and potential use as a laboratory tool for the investigation of patients with long COVID.

## Figures and Tables

**Table 1:** Detailed description of comparison subgroup analyses undertaken within this study.

Analysis 1. ANOVA test assessing CD4+ TCR vb family differences between nine overarching subgroups (HC-VAC, HC-UNVAC, MA (day 28), MA (6-month), MA (12-month), IP (day 7), IP (day 28), IP (6-month), and IP (12-month). PCA for assessment of subgroup clustering.
Analysis 2. ANOVA test assessing CD4+ TCR vb family differences between four COVID-19 patient groups (MA, IP moderate, IP severe, and IP critical, inclusive of all collected timepoints). PCA for assessment of subgroup clustering. Classification modelling attempt for subgroup separation (ANN).
Analysis 3. ANOVA test assessing CD4+ TCR vb family differences between three COVID-19 IP groups (IP moderate, IP severe, and IP critical, inclusive of all collected timepoints). PCA for assessment of subgroup clustering. Classification modelling attempt for subgroup separation (ANN).
Analysis 4. ANOVA test assessing CD4+ TCR vb family differences between ALL IP acute (day 7) vs. ALL IP convalescent (day 28, 6-month, 12-month). Severity groups combined together. PCA for assessment of subgroup clustering.
Analysis 5. ANOVA test assessing CD4+ TCR vb family differences between three COVID-19 IPs subgroups (moderate, severe, critical combined) during acute infection (day 7).
Analysis 6. ANOVA test assessing CD4+ TCR vb family differences between three COVID-19 IPs subgroups (moderate, severe, critical combined) during convalescence (day 28, 6 months, 12 months combined).
Analysis 7. ANOVA test (*p* < 0.05 considered statistically significant) assessing CD4+ TCR vb family differences between HC-VAC and HC-UNVAC cohorts. PCA for assessment of subgroup clustering.
Analysis 8. ANOVA test assessing CD4+ TCR vb family differences comparing HC-VAC with MA subgroup.
Analysis 9. ANOVA test assessing CD4+ TCR vb family differences comparing HC-VAC with IP patients at day 28 post infection. PCA for assessment of subgroup clustering.

**Table 2:** Laboratory features of participants at different stages of SARS-CoV-2 disease. *p*-values calculated based on an ANOVA test. * Statistically significant at a 95% confidence level (*p* < 0.05).

Laboratory Results Day 1					
Patient Group	Leukocyte × 10^9^/L mean (range) *p* = 0.975	Lymphocyte × 10^9^/L mean (range) *p* = 0.637	CD4^+^ T cells 10^6^/L mean (range) *p* = 0.941	CD8^+^ T cells 10^6^/L mean (range) *p* = 0.429	CRP mean (range) *p* = 0.725
HC Unvaccinated *n* = 38 Age = 42 (20–71) Male = 8 Female = 30	5.68 (3.27–9.37)	1.85 (0.89–3.26)	943.03 (410–1680)	452.03 (200–798)	2.1 (0.4–15.23)
HC Vaccinated *n =* 22 Age = 46 (25–65) Male = 3 Female = 19	5.67 (4.04–8.01)	1.78 (1.04–2.92)	936.67 (568–1810)	413.04 (147–777)	1.89 (0.11–5.44)
Laboratory Results Day 7					
Patient Group	Leukocyte × 10^9^/L mean (range) *p* = 0.107	Lymphocyte × 10^9^/L mean (range) *p* = 0.402	CD4^+^ T cells 10^6^/L mean (range) *p* = 0.826	CD8^+^ T cells 10^6^/L mean (range) *p* = 0.290	CRP mean (range) *p* = 0.867
In-Patient Moderate *n* = 2 Age = 72 (69–74) Male = 1 Female = 1	5.44 (4.6–6.27)	1.28 (1.04–1.52)	416 (337–495)	215.5 (182–249)	38.3 (17–59.6)
In-Patient Severe *n* = 6 Age = 66 (53–80) Male = 5 Female = 1	13.9 (11.5–17.64)	2.48 (1.03–5.15)	331.67 (202–561)	142.33 (86–208)	24.4 (9.1–76.3)
In-Patient Critical *n* = 9 Age = 62 (29–80) Male = 7 Female = 2	14.43 (8.2–23.08)	1.41 (0.24–2.13)	378.11 (118–720)	306.67 (42–801)	33.54 (1.2–129.7)
Laboratory Results Day 28					
Patient Group	Leukocyte × 10^9^/L mean (range) *p* = 0.607	Lymphocyte × 10^9^/L mean (range) *p* = 0.328	CD4^+^ T cells 10^6^/L mean (range) *p* = 0.050	CD8^+^ T cells 10^6^/L mean (range) *p* = 0.532	CRP mean (range) *p* = 0.271
Mild–Asymptomatic *n* = 26 Age = 42 (21–59) Male = 6 Female = 20	6.51 (3.78–9.86)	2.05 (1.35–3.81)	1029.07 (385–1439)	465.36 (107–890)	3.79 (0.28–16.33)
In-Patient Moderate *n* = 4 Age = 55 (39–74) Male = 2 Female = 2	5.56 (4.75–6.37)	1.31 (1.23–1.39)	764.5 (460–1106)	471.25 (279–623)	10.26 (1.17–33.79)
In-Patient Severe *n* = 12 Age = 59 (42–70) Male = 10 Female = 2	5.41 (4.14–6.7)	1.65 (1.26–2.2)	826.75 (661–1280)	445.08 (220–837)	7.27 (1–37.46)
In-Patient Critical *n* = 4 Age = 54 (48–67) Male = 3 Female = 1	7.18 (3.88–10.47)	1.62 (1.57–1.67)	678.25 (431–1006)	628.5 (114–1189)	9.5 (0.46– 26.1)
Laboratory Results 6 Months					
Patient Group	Leukocyte × 10^9^/L mean (range) *p* = 0.520	Lymphocyte × 10^9^/L mean (range) *p* = 0.109	CD4^+^ T cells 10^6^/L mean (range) *p* = 0.011 *	CD8^+^ T cells 10^6^/L mean (range) *p* = 0.294	CRP mean (range) *p* = 0.011 *
Mild–Asymptomatic *n* = 27 Age = 43 (21–59) Male = 6 Female = 21	7.57 (5.32–11.68)	2.6 (1.41–4.97)	1131.38 (576–3014)	493.59 (176–1109)	3.22 (0.34–13.63)
In-Patient Moderate *n* = 6 Age = 56 (31–74) Male = 2 Female = 4	6.5 (5.6–8.01)	1.69 (0.97–2.82)	518 (219–600)	732.17 (268–2127)	19.39 (2.28–75)
In-Patient Severe *n* = 11 Age = 58 (33–70) Male = 8 Female = 3	6.38 (4.98–7.16)	1.39 (0.62–2.11)	823.09 (227–1167)	481.64 (237–870)	6.33 (2.09–16.59)
In-Patient Critical *n* = 5 Age = 60 (48–79) Male = 3 Female = 2	6.43 (5.34–7.98)	1.67 (1.44–2)	799.4 (478–1315)	737.6 (221–1543)	1.8 (0.27–3.96)
Laboratory Results 12 Months					
Patient Group	Leukocyte × 10^9^/L mean (range) *p* = 0.698	Lymphocyte × 10^9^/L mean (range) *p* = 0.799	CD4^+^ T cells 10^6^/L mean (range) *p* = 0.114	CD8^+^ T cells 10^6^/L mean (range) *p* = 0.332	CRP mean (range) *p* = 0.001 *
Mild–Asymptomatic *n* = 26 Age = 43 (22–60) Male = 6 Female = 20	6.86 (4.17–11.36)	2.06 (1.02–4.08)	1030.89 (438–2243)	473.14 (176–1164)	3.02 (0.35–9.05)
In-Patient Moderate *n* = 4 Age = 52 (31–75) Male = 2 Female = 2	6.54 (5.32–7.48)	1.75 (1.07–2.52)	643.75 (508–814)	426.75 (291–727)	13.25 (7.3–19.19)
In-Patient Severe *n* = 12 Age = 59 (34–70) Male = 10 Female = 2	6.15 (4.35–7.97)	1.92 (1.13–2.41)	863.08 (357–1389)	427.25 (110–822)	4.13 (1.07–12.01)
In-Patient Critical *n* = 5 Age = 61 (49–80) Male = 3 Female = 2	6.54 (3.89–8.06)	1.78 (0.25–3.17)	817.4 (399–1420)	690 (93–1578)	1.35 (0.79–2.35)

**Table 3:** Frequency table of TCR vb family specificities demonstrating significant variance by ANOVA testing in group comparison experiments 1–9 (*p* < 0.05 considered statistically significant). Analysis 8 did not reveal any vb families with significant variance.

TCR Family	Analysis No.								Freq.
	1	2	3	4	5	6	7	9	
Vb 1					1				1
Vb 2					2				1
Vb 4		4	4			4	4		4
Vb 5.2	5.2	5.2	5.2	5.2		5.2			5
Vb 5.3		5.3							1
Vb 7.2				7.2					1
Vb 11		11						11	2
Vb 12	12	12	12			12			4
Vb 13.1	13.1	13.1		13.1		13.1			4
Vb 13.6		13.6							1
Vb 14	14	14							2
Vb 17		17	17		17	17			4
Vb 18	18	18		18				18	4
Vb 20	20	20	20	20		20		20	6
Vb 21.3		21.3							1
Vb 22				22					1

## Data Availability

The data underlying this article will be shared on reasonable request to the corresponding author.

## Supplementary Materials

The following supporting information can be downloaded at https://www.mdpi.com/article/10.3390/diagnostics14202330/s1, Figure S1: Exploratory analysis by PCA comparing in-patients at different time-points of acute (D7) COVID-19 disease and convalesence (D28, 6M, 12M) (a) ANOVA test for each TCR vb family (*p* < 0.05 is considered statistically significant [circled]). (b) PCA performed using the TCRvb families with *p* < 0.05. Some clustering separation between the D7 group can be observed. (c) Key loadings (features) on PC2; Table S1: Patient demographics; Table S2: TCR vB specificities within Beckman Coulter kit.
