# Supplementary material for: Investigation of Long-Term CD4+ T Cell Receptor Repertoire Changes Following SARS-CoV-2 Infection in Patients with Different Severities of Disease
Source: Diagnostics (Basel). 2024 Oct 19;14(20):2330. doi: 10.3390/diagnostics14202330 (PMC11507081; doi:10.3390/diagnostics14202330)
Supplement: Supplementary file 1 [file diagnostics-14-02330-s001.zip › diagnostics-3207705-supplementary.pdf]

Investigation of long-term CD4+ T cell receptor repertoire changes following SARS-COV-2 infection in patients with different severities of disease

<u>Supplemental files</u>

Results

Figure S1 (supplemental). Exploratory analysis by PCA comparing in-patients at different time-points of acute (D7) COVID-19 disease and convalesence (D28, 6M, 12M) a) ANOVA test for each TCR vb family (p < 0.05 is considered statistically significant [circled]). b) PCA performed using the TCR vb families with *p* < 0.05. Some clustering separation between the D7 group can be observed. c) Key loadings (features) on PC2.

a)

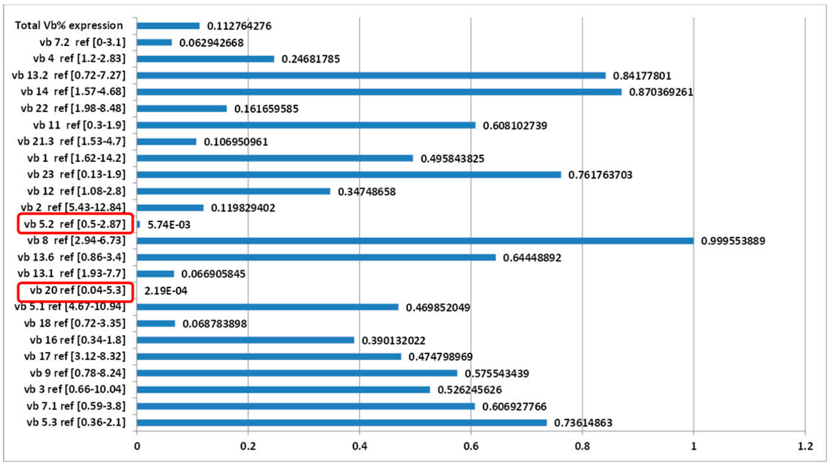

b)

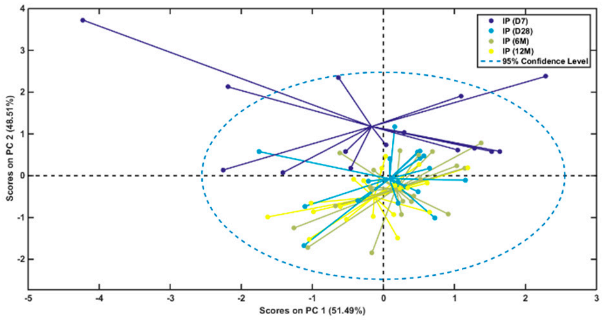

c)

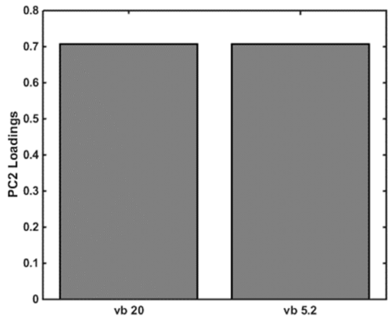

Supplemental Table S1. Patient demographics.

Patient Group	Study Number	Sex	Ethnicity	BMI	Number of Co-morbidities	Clinical Severity	GCS
Healthy Control (Unvaccinated)	RPH HC 001	M	White - European	23	0	N/A	15
Healthy Control (Unvaccinated)	RPH HC 002	F	White - British	20	1	N/A	15
Healthy Control (Unvaccinated)	RPH HC 003	M	White - British	29.4	0	N/A	15
Healthy Control (Unvaccinated)	RPH HC 004	M	White - British	26.1	0	N/A	15
Healthy Control (Unvaccinated)	RPH HC 005	M	White - British	24.9	0	N/A	15
Healthy Control (Unvaccinated)	RPH HC 007	M	White - British	30.5	0	N/A	15
Healthy Control (Unvaccinated)	RPH HC 008	F	White - British	41.5	1	N/A	15
Healthy Control (Unvaccinated)	RPH HC 009	F	White - British	24.9	1	N/A	15
Healthy Control (Unvaccinated)	RPH HC 010	F	Caribbean	26.4	0	N/A	15
Healthy Control (Unvaccinated)	RPH HC 011	F	White - British	22.1	1	N/A	15
Healthy Control (Unvaccinated)	RPH HC 012	F	White - British	28.7	0	N/A	15
Healthy Control (Unvaccinated)	RPH HC 013	F	White - British	27.6	1	N/A	15
Healthy Control (Unvaccinated)	RPH HC 014	F	African	27	1	N/A	15
Healthy Control (Unvaccinated)	RPH HC 015	F	White - British	23.6	3	N/A	15
Healthy Control (Unvaccinated)	RPH HC 017	F	White - British	32.6	1	N/A	15
Healthy Control (Unvaccinated)	RPH HC 018	F	Indian	27.6	0	N/A	15
Healthy Control (Unvaccinated)	RPH HC 019	F	White - British	35.2	2	N/A	15
Healthy Control (Unvaccinated)	RPH HC 020	F	White - British	29.8	0	N/A	15
Healthy Control (Unvaccinated)	RPH HC 021	F	White - British	41.4	3	N/A	15
Healthy Control (Unvaccinated)	RPH HC 022	F	White - British	22.2	1	N/A	15
Healthy Control (Unvaccinated)	RPH HC 023	F	White - British	29	0	N/A	15
Healthy Control (Unvaccinated)	RPH HC 024	M	White - British	24.5	0	N/A	15
Healthy Control (Unvaccinated)	RPH HC 025	F	White - British	38.1	0	N/A	15
Healthy Control (Unvaccinated)	RPH HC 026	F	White - British	25.5	0	N/A	15
Healthy Control (Unvaccinated)	RPH HC 027	F	White - British	24.9	1	N/A	15
Healthy Control (Unvaccinated)	RPH HC 029	F	White - British	25.9	0	N/A	15

Healthy Control (Unvaccinated)	RPH HC 030	M	White - British	21.5	3	N/A	15
Healthy Control (Unvaccinated)	RPH HC 031	F	White - British	26.5	0	N/A	15
Healthy Control (Unvaccinated)	RPH HC 032	F	White - British	24.1	0	N/A	15
Healthy Control (Unvaccinated)	RPH HC 033	M	White - British	25	0	N/A	15
Healthy Control (Unvaccinated)	RPH HC 034	F	White - British	26.5	2	N/A	15
Healthy Control (Unvaccinated)	RPH HC 035	F	White - British	27.5	0	N/A	15
Healthy Control (Unvaccinated)	RPH HC 036	F	Chinese	33.2	2	N/A	15
Healthy Control (Unvaccinated)	RPH HC 037	F	White - British	25.1	0	N/A	15
Healthy Control (Unvaccinated)	RPH HC 038	F	White - British	32.3	0	N/A	15
Healthy Control (Unvaccinated)	RPH HC 039	F	White - British	32.3	0	N/A	15
Healthy Control (Unvaccinated)	RPH HC 040	F	White - British	21.1	0	N/A	15
Healthy Control (Unvaccinated)	RPH HC 041	F	White - British	27.5	0	N/A	15

Healthy Control (Vaccinated)	RPH HC 042	F	White and Black Caribbean	21.8	1	N/A	15
Healthy Control (Vaccinated)	RPH HC 043	F	White - British	21.3	1	N/A	15
Healthy Control (Vaccinated)	RPH HC 044	F	White - British	23.2	2	N/A	15
Healthy Control (Vaccinated)	RPH HC 045	F	White - British	24.2	1	N/A	15
Healthy Control (Vaccinated)	RPH HC 046	F	White - British	35.4	3	N/A	15
Healthy Control (Vaccinated)	RPH HC 047	F	White - British	22.4	0	N/A	15
Healthy Control (Vaccinated)	RPH HC 048	F	White - British	30.1	1	N/A	15
Healthy Control (Vaccinated)	RPH HC 049	F	White - British	24.6	0	N/A	15
Healthy Control (Vaccinated)	RPH HC 050	F	White - British	36.8	1	N/A	15
Healthy Control (Vaccinated)	RPH HC 053	F	White - British	35.5	2	N/A	15
Healthy Control (Vaccinated)	RPH HC 054	F	White - British	22.8	0	N/A	15
Healthy Control (Vaccinated)	RPH HC 055	F	White - British	34.3	1	N/A	15
Healthy Control (Vaccinated)	RPH HC 056	M	White - British	25.5	0	N/A	15
Healthy Control (Vaccinated)	RPH HC 057	M	White - British	22.5	0	N/A	15
Healthy Control (Vaccinated)	RPH HC 058	M	White - British	24	0	N/A	15
Healthy Control (Vaccinated)	RPH HC 060	F	White - British	15.7	0	N/A	15

Healthy Control (Vaccinated)	RPH HC 061	F	White - British	22.5	0	N/A	15
Healthy Control (Vaccinated)	RPH HC 062	F	White - British	30.3	0	N/A	15
Healthy Control (Vaccinated)	RPH HC 063	F	Indian	25.9	0	N/A	15
Healthy Control (Vaccinated)	RPH HC 064	F	White - British	26.5	0	N/A	15
Healthy Control (Vaccinated)	RPH HC 065	F	White - British	31.5	1	N/A	15
Healthy Control (Vaccinated)	RPH HC 066	F	White - British	31.4	0	N/A	15

Mild-Asymptomatic	RPH MA 001	F	White - British	23.3	0	N/A	15
Mild-Asymptomatic	RPH MA 002	F	-	27.4	-	N/A	15
Mild-Asymptomatic	RPH MA 003	F	White - British	30.8	0	N/A	15
Mild-Asymptomatic	RPH MA 004	F	White - British	33.4	0	N/A	15
Mild-Asymptomatic	RPH MA 005	F	White - British	22.2	0	N/A	15
Mild-Asymptomatic	RPH MA 006	F	White - British	25.2	0	N/A	15
Mild-Asymptomatic	RPH MA 007	F	White - British	30.4	1	N/A	15
Mild-Asymptomatic	RPH MA 008	M	White - British	33.2	1	N/A	15
Mild-Asymptomatic	RPH MA 009	F	White - British	24.1	0	N/A	15
Mild-Asymptomatic	RPH MA 010	F	White - British	27	0	N/A	15
Mild-Asymptomatic	RPH MA 011	F	White - British	33.5	0	N/A	15
Mild-Asymptomatic	RPH MA 012	F	White - British	34.4	0	N/A	15
Mild-Asymptomatic	RPH MA 013	M	White - British	26.8	1	N/A	15
Mild-Asymptomatic	RPH MA 014	M	White - British	22.7	0	N/A	15
Mild-Asymptomatic	RPH MA 015	F	White - British	27.2	0	N/A	15
Mild-Asymptomatic	RPH MA 016	F	White - British	30.3	0	N/A	15
Mild-Asymptomatic	RPH MA 017	F	White - British	24.5	2	N/A	15
Mild-Asymptomatic	RPH MA 018	F	White - British	34.7	2	N/A	15
Mild-Asymptomatic	RPH MA 019	F	White - British	31.1	0	N/A	15
Mild-Asymptomatic	CDH MA 001	F	White - British	24	0	N/A	15
Mild-Asymptomatic	CDH MA 002	M	White - British	30.4	1	N/A	15
Mild-Asymptomatic	CDH MA 003	F	White and Asian	32.2	0	N/A	15

Mild-Asymptomatic	CDH MA 004	F	White - British	25.9	0	N/A	15
Mild-Asymptomatic	CDH MA 005	F	White and Asian	22.1	1	N/A	15
Mild-Asymptomatic	CDH MA 006	F	White - British	38.6	1	N/A	15
Mild-Asymptomatic	CDH MA 007	M	White - British	27.1	2	N/A	15
Mild-Asymptomatic	CDH MA 008	F	White - British	31.3	1	N/A	15

In-Patient	RPH IP 002	M	White - British	45.1	0	Severe	15
In-Patient	RPH IP 003	M	White - British	26.1	1	Critical	15
In-Patient	RPH IP 004	F	White - British	28.3	1	Critical	15
In-Patient	RPH IP 005	F	White - British	25.3	4	Moderate	15
In-Patient	RPH IP 006	M	Indian	29.5	5	Critical	15
In-Patient	RPH IP 007	M	White - British	32.3	0	Moderate	15
In-Patient	RPH IP 008	M	White - British	21.7	2	Severe	15
In-Patient	RPH IP 009	F	White - British	31.8	1	Critical	15
In-Patient	RPH IP 010	F	White - British	28.6	3	Severe	15
In-Patient	RPH IP 011	F	Pakistani	30.2	7	Severe	15
In-Patient	RPH IP 012	M	White - British	23.9	4	Severe	15
In-Patient	RPH IP 013	M	Indian	37	2	Moderate	15
In-Patient	RPH IP 014	M	White - British	31	1	Severe	15
In-Patient	RPH IP 015	M	White - British	30	1	Severe	15
In-Patient	RPH IP 016	M	White - British	30.4	2	Severe	15
In-Patient	RPH IP 017	M	White - British	30.4	3	Critical	15
In-Patient	RPH IP 018	M	White - British	37.1	0	Severe	15
In-Patient	RPH IP 019	M	White - British	29.3	1	Critical	15
In-Patient	RPH IP 020	M	White - British	40.1	2	Severe	15
In-Patient	RPH IP 021	M	White - British	26.7	0	Critical	15
In-Patient	RPH IP 022	M	White - British	25.3	4	Severe	15
In-Patient	RPH IP 023	M	White - British	26	0	Critical	15
In-Patient	RPH IP 024	M	White - British	16.3	3	Severe	15

In-Patient	RPH IP 025	M	White - British	28.3	2	Critical	15
In-Patient	RPH IP 026	M	White - British	30.3	1	Severe	15
In-Patient	RPH IP 027	F	White - British	29.4	0	Severe	15
In-Patient	RPH IP 028	M	White - British	30	3	Severe	15
In-Patient	RPH IP 029	M	White - British	32.2	1	Severe	15
In-Patient	RPH IP 030	M	White - British	27.8	4	Moderate	15
In-Patient	RPH IP 031	F	White - British	40	1	Critical	15
In-Patient	RPH IP 032	M	White - British	24.3	2	Severe	15
In-Patient	RPH IP 033	M	White - British	29	1	Critical	15
In-Patient	RPH IP 034	M	White - British	29.8	2	Critical	15
In-Patient	RPH IP 035	F	White - British	17.1	0	Moderate	15
In-Patient	RPH IP 036	M	White - British	24.6	1	Critical	15
In-Patient	RPH IP 037	F	White - British	34.2	4	Moderate	15
In-Patient	CDH IP 001	M	Other	25.2	2	Moderate	15
In-Patient	CDH IP 002	M	White - British	22.3	5	Severe	15
In-Patient	CDH IP 003	F	White - British	24.3	2	Moderate	15

BMI - Body Mass Index; GCS – Glasgow Coma Score.

Table S2 (supplemental). TCR vb specificities within Beckman Coulter kit.

Tube	Clone	Conjugate	Specificity	Isotype
**A**	3D11	PE	VB 5.3	IgG1 (mouse)
	ZOE	FITC + PE	VB 7.1	IgG2a (mouse)
	CH92	FITC	VB 3	IgM (mouse)
**B**	FIN9	PE	VB 9	IgG2a (mouse)
	E17.5F3.15.13	FITC + PE	VB 17	IgG1 (mouse)
	TAMAYA1.2	FITC	VB 16	IgG1 (mouse)
**C**	BA62.6	PE	VB 18	IgG1 (mouse)
	IMMU157	FITC + PE	VB 5.1	IgG2a (mouse)
	ELL1.4	FITC	VB 20	IgG (mouse)
**D**	IMMU222	PE	VB 13.1	IgG2b (mouse)
	JU74.33	FITC + PE	VB 13.6	IgG1 (mouse)
	56C5.2	FITC	VB 8	IgG2a (mouse)
**E**	36213	PE	VB 5.2	IgG1 (mouse)
	MPB2D5	FITC + PE	VB 2	IgG1 (mouse)
	VER2.32.1.1	FITC	VB 12	IgG2a (mouse)
**F**	AF23	PE	VB 23	IgG1 (mouse)
	BL37.2	FITC + PE	VB 1	IgG1 (rat)
	IG125	FITC	VB 21.3	IgG2a (mouse)
**G**	C21	PE	VB 11	IgG2a (mouse)
	IMMU546	FITC + PE	VB 22	IgG1 (mouse)
	CAS1.1.3	FITC	VB 14	IgG1 (mouse)
**H**	H132	PE	VB 13.2	IgG1 (mouse)
	WJF24	FITC + PE	VB 4	IgM (rat)
	ZIZOU4	FITC	VB 7.2	IgG2a (mouse)
